# Healthcare Staff Views on the Roles of Psychologists in Hospital Care: A Survey in Italy

**DOI:** 10.1007/s10880-026-10142-3

**Published:** 2026-04-16

**Authors:** Lorenza Magliano, Gaetana Affuso, Alda Troncone, Michele Orditura, Zuzana Simonova

**Affiliations:** 1 Department of Psychology, University of Campania “Luigi Vanvitelli”, Caserta, Italy; 2 Department of Precision Medicine, Naples, University of Campania “Luigi Vanvitelli”, Caserta, Italy; 3 UOC Medical Oncology, Azienda Ospedaliera di Rilievo Nazionale e di Alta Specializzazione (AORN) Sant’Anna e San Sebastiano, Caserta, Italy; 4 UOC Preventive Medicine, Occupational Medicine, and Radiation Protection, Azienda Ospedaliera di Rilievo Nazionale e di Alta Specializzazione (AORN) Sant’Anna e San Sebastiano, Caserta, Italy

**Keywords:** Hospital care, Psychology, Healthcare staff, Views

## Abstract

Despite benefits for patients, families, and healthcare professionals, psychology remains only partially integrated into hospital care. A brief, practical questionnaire for routine use could help understand staff perceptions and support psychologists’ integration. This study aimed to (a) develop and validate the Psychologist in Hospital Questionnaire (PHQ), an 18-item self-administered Italian instrument designed to assess staff perceptions of psychologists’ roles in three domains: support for patients and families, integration into care pathways, and support and training for staff; (b) examine whether domain scores reflect professional and organizational differences. Between December 2024 and May 2025, all healthcare professionals were invited to complete the PHQ in a general hospital in Southern Italy. The instrument’s psychometric structure was tested via confirmatory factor analysis (CFA), and its internal consistency was assessed with Cronbach’s *α*. Differences in PHQ scores across professional and organizational variables were examined. CFA, performed on a sample of 347 participating staff, supported the three-factor structure with good fit indices and high internal consistency (*α* = 0.80–0.89). Psychologists were most valued for supporting and training staff, followed by supporting patients and families. Greater awareness of psychologists’ support was observed among staff in units with regular access to psychologists and in medical units, supporting construct validity. The PHQ is a reliable 18-item tool suitable for routine evaluation of staff perceptions of psychologists’ roles. Its use may guide hospital service planning and foster organizational models that strengthen psychologists’ presence in healthcare teams.

## Introduction

In recent years, the growing focus on mental health has contributed to redefining the role of psychology within healthcare systems. The COVID-19 pandemic has accelerated this process, highlighting the need to address psychological well-being as an integral part of public health and promoting a multidisciplinary approach to care (World Health Organization, 2022). Within this framework, psychologists are requested to meet the emotional, relational, and communicative needs of patients, their families, and healthcare teams (Leventhal et al., 2021; Robiner & Petrik, 2017). These aspects are particularly relevant in hospital settings, where the management of complex clinical situations can be stressful for all parties involved (Palmer et al., 2025).

Studies have shown that integrating psychologists into care pathways improves clinical outcomes, enhances treatment adherence, and contributes to containing healthcare costs (Kidd & Styron, 2020; Sumner et al., 2018). Psychologists’ inclusion in multidisciplinary teams also contributes to burnout prevention, a better work environment, and improved conflict management (Robiner & Petrik, 2017; Palmer, 2025; Agapoff et al., 2020; Yost et al., 2022; West et al., 2016; Shanafelt et al., 2019). From the patient’s perspective, psychologists support care satisfaction and adjustment to illness (Bullock et al., 2022). Despite these benefits, psychologists in hospital settings are often involved only in specific situations, such as bereavement, adverse diagnoses, or psychiatric comorbidities (Kidd & Styron, 2020; Leventhal et al., 2021; Fann et al., 2008). Their integration into care teams remains limited (Palmer et al., 2025; Tomai et al., 2017), partly due to being considered by medical staff as peripheral figures, useful only for emotionally vulnerable patients (Feldman et al., 2025; Kidd & Styron, 2020; Tomai et al., 2017).

This marginalization is reinforced by limited awareness of the psychologists’ role in healthcare and confusion with the role of psychiatrists (Kidd & Styron, 2020; Takagishi et al., 2024; Leventhal et al., 2021; Palmer et al., 2025). Doctors and nurses also differ in perception: the former see psychologists as necessary for dealing with severe distress, while the latter more often recognize their broader value (Schwarzkopf et al., 2024; Small & Gault, 1975). Stigma remains a barrier. Patients may reject psychological support fearing negative judgments (Corrigan & Rao, 2012). Those with pre-existing psychiatric comorbidities are sometimes viewed by the staff as more difficult to manage and are treated differently from other patients (Shefer et al., 2014; Magliano et al., 2017; Pasere et al., 2025; Foye et al., 2020). Likewise, staff often avoid seeking help so as to protect their professional image (Garelick, 2012; Wang et al., 2025; Badford et al., 2023).

A true integration of psychology into hospital care therefore requires an understanding of how healthcare staff perceive the psychologist, not just as a clinical resource for patients, but also as a team member and promoter of workplace well-being. Existing research on this topic has primarily focused on clinical and consulative aspects, often relying on small samples, qualitative methodology, and non-validated tools. Moreover, studies have mainly focused on the views of medical staff, neglecting the perspectives of nurses, allied professionals, and care assistants (Palmer et al., 2025; Tomai et al., 2017; Leventhal et al., 2021; Kidd & Styron, 2020). While these studies offer valuable insights, their methodological limitations make it difficult to generalize findings or apply them systematically in hospital practice.

In light of the above considerations, in 2024, the Department of Psychology of the University of Campania “Luigi Vanvitelli”, Caserta, Italy conducted a research program at the Sant’Anna e San Sebastiano General Hospital in Caserta (Italy) involving all healthcare staff. The program comprised two distinct studies: the first study investigated healthcare professionals’ views on the roles of psychologists in hospital settings, while the second study explored staff attitudes toward the management of patients with psychiatric comorbidities and the potential contribution of psychologists in that management. Data for both studies were collected concurrently from the same sample, yet the two studies addressed different research questions and used different assessment tools.

To achieve the objectives of the first study, the research team developed the Psychologist in Hospital Questionnaire (PHQ), a new instrument designed to assess hospital staff perceptions of psychologists’ roles across three domains: support for patients and families, integration into care pathways, and support and training for staff. The present paper reports on the development and psychometric validation of this instrument. Specifically, the study aimed to

1. evaluate the main psychometric properties of the PHQ, including content validity, internal consistency, and construct validity and
2. explore whether PHQ domain scores vary according to organizational and professional characteristics—such as department type, professional role, or the presence of psychologists in the unit—as indicators of the instrument’s sensitivity to contextual differences.

## Methods

### Setting and Study Design

The study was conducted at the Sant’Anna e San SebastianoGeneral Hospital in Caserta (Italy), between December 2024 and May 2025. This hospital contains eight clinical departments, coordinated by a strategic management unit. Out of 1823 employees, 1631 are healthcare or social care professionals (AORN, 2024). Psychological support is available on request through the Preventive Medicine Service (one psychologist). The oncology and pediatrics units have psychologists for time-slot assistance to patients, while geriatrics and neurology units have temporary research psychologists for neuropsychological assessment of dementia.

All healthcare workers were invited to participate anonymously in the online Survey on Psychology and Psychiatric Comorbidity in Hospitals (SPPH). The SPPH includes three distinct self-administered questionnaires exploring staff views on (a) the roles of psychologists in hospital settings (Psychologists in Hospital Questionnaire—PHQ, study 1) and (b–c) the management of psychiatric comorbidity in hospital settings (Psychiatric Comorbidity in Hospital Questionnaire—PCHQ) and the involvement of psychologists in the management of psychiatric comorbidity in hospital settings (Psychologists in Psychiatric Comorbidity in Hospital Questionnaire—PPCHQ; study 2).

### Data Collection Procedures

Following authorization from hospital management, the project was presented to the heads of the departments via an informative e-mail containing a direct link to the online SPPH. The study was subsequently introduced in the departments by the research team, including the hospital psychologist. On these occasions, department heads or their delegates were asked to display the participation invitation, including the QR code of the questionnaire, in areas accessible to healthcare and social care personnel. In addition, paper copies of the SPPH were distributed by healthcare staff coordinators in the late recruitment phase and later collected and entered in the online database by the research team.

To encourage participation, the hospital preventive medicine unit sent out an initial e-mail to all healthcare personnel, containing the SPPH link and QR code, followed by three reminder e-mails, also including updates on participation rates. Moreover, the research team sent WhatsApp reminders with the SPPH link to healthcare staff coordinators and physicians, inviting them to share them with colleagues. Participants completed the questionnaire using their personal electronic devices. All questions were optional, except for those required to provide informed consent for data processing and publication purposes. Respondents could at any time abandon filling out the questionnaire by closing the link page. The answers already given were thus automatically deleted.

### Instrument Development

The PHQ is a self-report questionnaire developed ad hoc with reference to the Italian “Consensus Document on Hospital Psychology” (FISP, 2013), the Italian “Guidelines on the Role of Psychology in the National Health Service” (Ministry of Health, 2022a, 2022b), and the APA’s “Guidelines for Psychology in Health Care Delivery Systems” (APA, 2013). These documents outline the strategic role of psychology in healthcare. The 2013 Consensus Document further highlights the evidence-based contributions of hospital psychologists to patient care and organizational processes. In the hospital setting, the 2022 national guidelines assign psychology a coordinating and integrative function across clinical and organizational activities, aimed at supporting patients, families, staff and the overall quality and humanization of care. The APA Practice Guidelines were also used as a general reference for the core functions of the psychology profession. Together, these documents formed the conceptual basis of the questionnaire. The instrument was originally developed in Italian. Based on these documents, one member of the research team (LM) drafted a preliminary list of questions, which was subsequently discussed and revised with a second member (AT). This process led to an 18-item version of the tool. Face validity was assessed following recommended procedures for early-stage instrument development (Boateng et al., 2018). The preliminary version was evaluated for clarity and comprehensibility by ten healthcare professionals from various disciplines external to the hospital where the study was conducted (four physicians, two nurses, one healthcare assistant, one psychologist, one physiotherapist, and one midwife; five women, seven aged > 54 years, two aged 30–44 years, and one aged 45–54 years). Each item was rated on a 6-point scale (1 = “not at all clear/incomprehensible” to 6 = “completely clear/comprehensible”). All items received mean scores above 5 (Table 2) and were therefore retained in this version. The instrument was then reviewed and approved by the hospital psychologist (ZS), who confirmed the adequacy and relevance of all items. The final version of the PHQ comprises 18 items exploring the respondents’ views regarding the role of psychologists in the following three domains: (1) support for patients and families (7 items): focuses on psychological and relational support during serious or poor prognosis illnesses, including the emotional adaptation of patients and their families; (2) integration into care pathways (5 items): examines the psychologist’s involvement in interdisciplinary care planning and collaboration with the medical team; and (3) support and training for healthcare staff (6 items): explores psychologists’ contributions to team well-being, including emotional support and staff training. Items were rated on a 6-point Likert scale (from “not really true” to “really true”). A short final section collects respondents’ socio-demographic and professional information. Respondents were invited to answer with reference to “a unit like the one where I work,” or, if not working in a specific clinical unit, to respond about their experience in a hospital context. The questionnaire also included one open-ended item inviting additional comments on psychologists’ roles; these qualitative responses were not analyzed in the present paper, which focuses on the psychometric and quantitative findings. The full Italian version of the PHQ is available from the corresponding author upon reasonable request.

### Statistical Analysis

Frequencies and means (± SD), as appropriate, were computed for the main participants’ socio-demographic and professional characteristics and for each of the PHQ’s 18 items. Confirmatory Factor Analysis (CFA) was performed to verify the construct validity of the PHQ items, using maximum likelihood estimation of the covariance matrix. Several indexes were used to determine model fit: the comparative fit index (CFI), the Tucker–Lewis index (TLI), the root mean square error of approximation (RMSEA), and the standardized root mean square residual (SRMR). A CFI and TLI ≥ .90 and an SRMR and RMSEA ≤ .08 indicate a model’s acceptable fit to the data (Hu & Bentler, 1999). Cronbach’s *α* coefficients were calculated to assess the internal consistency reliability of the factors confirmed by CFA. Means (± SD) were computed for the PHQ subscales. Repeated-measures Analysis of Variance (ANOVA) with Bonferroni post hoc test was used to explore differences between subscale mean scores. Pearson’s correlation coefficients were calculated to examine relationships between PHQ subscale mean scores and participants’ age, years of professional experience, and length of employment in the hospital. Multivariate Analysis of Variance (MANOVA) with Bonferroni post hoc test was applied to test differences in PHQ subscale mean scores (dependent variables) according to department, unit type, professional role and regular presence of psychologist in the unit (independent variables; details in Table 1). When calculating subscale mean scores, missing data in individual items were replaced by the mean of valid responses for that subscale. Missing data on socio-demographic and professional variables were not replaced, and multivariate analyses were therefore conducted separately for each independent variable. Statistical significance level was set at *p* < .05. CFA was performed using Mplus version 7.4 (Muthén & Muthén, 2017). All other analyses were conducted using IBM SPSS Statistics, version 21.

**Table 1:** Participants’ socio-demographic and professional characteristics (*N* = 347)

Variable	Value
Age (years), mean ± SD (missing)	45.2 ± 10.9 (25)
Gender, *N* (%)	
Male	123 (36.5)
Female	214 (63.5)
Missing	10
Professional role, *N* (%)	
Physician	88 (27.3)
Nurse	160 (49.7)
Other health professional	31 (9.6)
Health care assistant	43 (13.4)
Missing	25
Years of work in health field, mean ± SD (missing)	18.3 ± 10.9 (43)
Years of work in Caserta hospital, mean ± SD (missing)	11.5 ± 10.9 (47)
Department, *N* (%)	
Cardiovascular	32 (10.9)
Health services^a^	14 (4.8)
Women and children	38 (13.0)
Emergency and admissions	54 (18.4)
Oncology and hematology	21 (7,2)
Surgical sciences	27 (9.2)
Medical sciences	81 (27.6)
Head and neck^b^	20 (6.8)
Health management	6 (2.0)
Missing	54
Type of unit, *N* (%)	
Medical	151 (51.5)
Surgical	82 (28.0)
Support services	60 (20.5)
Missing	54

^a^Includes the units: diagnostic imaging, analysis laboratory, nuclear medicine, microbiology and virology, and transfusion medicine service

^b^Includes the units: maxillofacial surgery, neurosurgery, ophthalmology, and otolaryngology

## Results

### Participant Characteristics

A total of 358 questionnaires were collected (273 online and 85 on paper), with a response rate of 21.9%. Eleven questionnaires were excluded (nine completed by technical staff and two left blank), leaving a final sample of 347 participants. Respondents were predominantly nurses or physicians, with over ten years of work experience and primarily employed in medical units. There was a large representation of women among respondents (Table 1). Seventy-four percent (*n* = 325) reported that no psychologist was available in their unit, 15.7% indicated availability on request, and 10.1% reported a psychologist regularly present for some days/every day in the unit.

### Construct Validity and Internal Consistency of the PHQ

Confirmatory Factor Analysis (CFA) confirmed the three-factor structure of the PHQ (Table 2): support to patients and families (factor 1), integration into care pathways (factor 2) and training and support to multidisciplinary staff (factor 3). The final model demonstrated a good fit to the data, *χ*^2^(123) = 359.37, *p* < 0.001; Tucker–Lewis Index (TLI) = 0.92; Comparative Fit Index (CFI) = 0.93; Root Mean Square Error of Approximation (RMSEA) = 0.074, 90% CI [0.066, 0.083]; and Standardized Root Mean Square Residual (SRMR) = 0.047. All factor loadings were significant at *p* < 0.001. The correlations among the three factors were all significant (factor 1 with factor 2: *β* = .95, *p* < *.*001; factor 1 with factor 3: *β* = .86, *p* < .001; factor 2 with factor 3: *β* = .88, *p* < .001—standardized solution). Internal consistency reliability was high, with Cronbach’s *α* coefficients ranging from 0.80 to 0.89 across the three domains (Table 2). These results support the construct validity and internal consistency of the PHQ.

**Table 2:** Psychometric analysis on the PHQ items: clarity/comprehensibility and confirmatory factor analysis

Items	Clarity/comprehensibility (*N* = 10)	Factor loadings (*N* = 347)		
	Mean (SD)	1	2	3
1. Assess the mental state of patients*	5.6 (0.97)	0.56		
2. Administer tests for the diagnosis of mental disorders*	5.9 (0.32)	0.53		
3. Help patients adapt to their illness*	5.8 (0.42)	0.70		
4. Support the families of patients with serious diseases*	5.9 (0.32)	0.66		
5. Accompany patients in making ethical decisions (e.g., living wills, therapeutic abortion)*	6.0 (0)	0.71		
6. Collaborate in the management of serious diseases (e.g., cancer, heart disease)*	5.9 (0.32)		0,70	
7. Promote a good relationship between doctors and patients*	5.6 (0.84)		0,77	
8. Collaborate in the development of care plans*	5.8 (0.63)		0.73	
9. Facilitate the active participation of patients in decisions about their care*	5.5 (1.08)		0.71	
10. Provide health education for patients (e.g., proper nutrition, physical activity)*	5.6 (0.84)		0.58	
11. Maintain relationships with patient and family associations and social volunteers*	5.7 (0.67)		0.57	
12. Support healthcare staff in caring for patients with a poor prognosis*	5.8 (0.63)			0.78
13. Support medical staff in communicating a poor prognosis to patients*	5.8 (0.63)			0.75
14. Support medical staff in communicating the death of a patient to family members*	5.9 (0.32)			0.64
15. Take care of the mental well-being of healthcare personnel*	5.7 (0.48)			0.74
16. Train healthcare personnel in managing work-related stress*	5.6 (0.84)			0.62
17. Train healthcare personnel in managing aggression from users*	5.4 (0.97)			0.68
18. Facilitate communication between different professionals*	5.5 (0.97)			0.72
	Cronbach’s *α*	0.80	0.84	0.89

The asterisk (*) indicates “In a unit like the one where I work, a psychologist should.” Factor 1: Support to patients and families; factor 2: Integration into care pathways; factor 3: Training and Support to multidisciplinary staff

The item content is translated from Italian

### Descriptive Results on PHQ Items

Most respondents (90%) agreed or strongly agreed (scores 5–6) with the statement that “Psychologists should be responsible for the well-being of staff,” and 87% endorsed the importance of “training staff on how to manage work-related stress” (Table 3). Eighty-five percent agreed that “Psychologists should support the families of patients with serious illnesses,” and 81% indicated that “Psychologists should assist other professionals in caring for patients with a poor prognosis.” Eighty percent agreed that “Psychologists should help patients adapt to their illness.” Seventy-five percent of respondents agreed that “Psychologists should promote a good relationship between doctors and patients,” and 62% believed that “Psychologists should encourage patients to participate actively in decisions about their care.”

**Table 3:** Staff views on the role of psychologists in hospital units (*N* = 347)

PHQ items	Not really true really true											
	1		2		3		4		5		6	
	*N*	%	*N*	%	*N*	%	*N*	%	*N*	%	*N*	%
Support for patients and families												
Assess the mental state of patients^a^	20	5.8	2	1.2	24	7.0	42	12.2	71	20.7	182	53.1
Administer tests for the diagnosis of mental disorders^a^	32	9.4	28	8.2	34	10.0	64	18.8	64	18.8	119	34.9
Help patients adapt to their illness^a^	13	3.8	4	1.2	17	5.0	34	10.0	80	23.5	192	56.5
Support the families of patients with serious diseases^a^	7	2.0	2	0.6	12	3.5	31	9.0	66	19.1	228	65.9
Accompany patients in making ethical decisions (e.g., living wills, therapeutic abortion)^a^	21	6.2	11	3.2	24	7.1	40	11.8	86	25.3	158	46.5
Integration into care pathways												
Collaborate in the management of serious diseases (e.g., cancer, heart disease)^a^	17	5.0	6	1.8	14	4.2	31	9.2	68	20.2	201	59.6
Promote a good relationship between doctors and patients^a^	13	3.8	7	2.1	25	7.3	39	11.4	85	24.9	172	50.4
Collaborate in the development of care plans^a^	25	7.5	25	7.5	39	11.6	54	16.1	81	24.2	111	33.1
Facilitate the active participation of patients in decisions about their care^a^	19	5.6	12	3.6	34	10.1	62	18.3	75	22.2	136	40.2
Provide health education for patients (e.g., proper nutrition, physical activity)^a^	31	9.2	19	5.6	44	13.0	52	15.4	78	23.1	114	33.7
Maintain relationships with patient and family associations and social volunteers^a^	18	5.3	20	5.8	42	12.3	65	19.0	77	22.5	120	35.1
Support and training for healthcare staff												
Support medical staff in communicating a poor prognosis to patients^a^	10	2.9	10	2.9	19	5.6	47	13.9	67	19.8	186	54.9
Support healthcare staff in caring for patients with a poor prognosis^a^	6	1.8	9	2.6	11	3.2	39	11.4	74	21.7	202	59.2
Support medical staff in communicating the death of a patient to family members^a^	12	3.5	10	2.9	14	4.1	35	10.2	73	21.3	198	57.9
Take care of the mental well-being of healthcare personnel^a^	4	1.2	7	2.0	8	2.3	16	4.6	61	17.7	249	72.2
Train healthcare personnel in managing work-related stress^a^	6	1.7	3	0.9	13	3.8	23	6.6	62	17.9	239	69.1
Train healthcare personnel in managing aggression from users^a^	9	2.6	2	0.6	16	4.6	27	7.8	78	22.6	213	61.7
Facilitate communication between different professionals^a^	9	2.6	6	1.7	13	3.8	43	12.5	83	24.1	191	55.4

^a^In a unit like the one where I work, a psychologist should

The item content is translated from Italian

### Analyses Performed on PHQ Subscales

Repeated-measures ANOVA revealed significant differences between PHQ subscale mean scores, *F* (2, 696) = 90.18, *p* < 0.001, partial *η*^2^ = 0.206, Mauchly’s sphericity *W* = 0.993, *p* = 0.30. Psychologists were perceived as most relevant in support and training for staff (*M* = 5.29, SD = 0.90), followed by support for patients and families (*M* = 4.96, SD = 1.02) and integration into care pathways (*M* = 4.71, SD = 1.10). Pairwise comparisons with Bonferroni correction indicated that all differences between subscales were statistically significant (all *p* < 0.001).

MANOVA revealed significant effects of department, unit type, and psychologist availability in the unit on PHQ scores. By department (Wilks’ *λ* = 0.86, *F*(3, 24) = 1.76, *p* < 0.05; Table 4), significant differences emerged in perceptions of support for patients and families (*F*(8) = 3.17, *p* < 0.002), with staff in oncology reporting higher scores than those in head–neck department (including maxillofacial surgery, neurosurgery, ophthalmology, and otolaryngology units) and health services department (including diagnostic imaging, analysis laboratory, nuclear medicine, microbiology and virology, and transfusion medicine units; Bonferroni *p* < 0.05). By unit type (Wilks’ *λ* = 0.94, *F*(3, 6) = 3.19, *p* < 0.01), significant differences were found in support for patients and families (*F*(2) = 6.17, *p* < 0.01) and integration into care pathways (*F*(2) = 4.25, *p* < 0.05), with medical-unit staff showing greater recognition of psychologists’ roles compared to those in support services (Bonferroni *p* < 0.01 and *p* < 0.05, respectively). By psychologist availability (Wilks’ *λ* = 0.96, *F*(3, 6) = 3.77, *p* < 0.01), staff from units with a regularly present psychologist reported higher scores for support for patients and families (*F*(2) = 7.23, *p* < 0.01). Acknowledgment of psychologists in the support for patients and their families was higher among respondents with fewer years of experience in healthcare (*r* = − 0.11, *p* < 0.05) and the hospital setting (*r* = − .12, *p* < 0.04).

**Table 4:** PHQ subscale scores across professional and organizational groups (MANOVA)

	PHQ subscales			
	Support for patients and families	Integration into care pathways	Support and training for healthcare staff	MANOVA
	Mean (SD)	Mean (SD)	Mean (SD)	Wilks *λ*, *F* (df), *p* <
Department (*N*)				
Cardiovascular (32)	5.00 (1.22)	4.85 (1.24)	5.38 (1.24)	
Health services (14)	4.53 (1.10)*^,^^b^	4.57 (1.00)	5.39 (0.89)	
Women and children (38)	4.80 (1.01)	4.69 (0.89)	5.16 (0.87)	
Emergency and admissions (54)	4.88 (1.05)	4.60 (1.31)	5.43 (0.69)	
Oncology and hematology (21)	5.61 (0.53)*^,^^a^	5.09 (0.92)	5.64 (0.67)	
Surgical sciences (27)	5.17 (0.76)	4.73 (0.92)	5.37 (0.64)	
Medical sciences (81)	5.19 (0.73)	4.92 (0.87)	5.36 (0.68)	
Head and neck (20)	4.64 (1.02)*^,^^b^	4.50 (0.92)	4.98 (1.11)	
Health management (6)	4.40 (0.59)	4.03 (0.73)	4.71 (0.82)	
* F* (df), *p* <	3,17 (8),0.01	1,32 (8),0.24	1,58 (8) 0.13	0.86, 1.76 (3, 24), 0.05
Unit (*N*)				
Medical (151)	5.17 (0.88)*^,^^a^	4.92 (0.95)*^,^^a^	5.43 (0.81)	
Surgical (82)	4.93 (0.92)	4.63 (0.92)	5.17 (0.88)	
Support services (60)	4.69 (1.08)*^,^^b^	4.51 (1.28)*^,^^b^	5.30 (0.81)	
* F* (df), *p* <	6.17 (2), 0.01	4.55 (2), 0.05	2.83 (2), 0.06	0.94, 3.19 (3, 6), 0.01
Professional role				
Physician (88)	5.12 (0.85)	4.68 (0.99)	5.30 (0.83)	
Nurse (160)	4.85 (1.05)	4.68 (1.12)	5.24 (0.96)	
Health care assistant (43)	5.09 (1.08)	4.86 (1.16)	5.45 (0.72)	
Other health professional (31)	5.07 (0.72)	4.72 (0.79)	5.43 (0.58)	
* F* (df), *p* <	1.89 (3), 0.13	0.32 (3), 0.81	1.06 (3), 0.37	0.96, 1.31 (3, 9), 0.22
Units with regular access to psychologists (*N*)				
Not available (254)	4.94 (0.97)	4.75 (1.05)	5.31 (0.85)	
Available (39)	5.38 (0.72)	4.80 (0.91)	5.45 (0.770	
* F* (df), *p* <	7.24 (1), 0.01	0.10 (1), 0.75	0.89 (1), 0.35	0.96, 3.77 (3), 0.01

*Bonferroni post hoc comparisons: *a* > *b*

## Discussion

This study provides both psychometric and empirical evidence supporting the Psychologist in Hospital Questionnaire (PHQ) as a valid and reliable instrument for assessing healthcare staff’s perceptions of psychologists’ roles in hospital settings. The confirmatory factor analysis confirmed a clear three-factor structure—support for patients and families, integration into care pathways, and support and training for staff—with excellent fit indices and high internal consistency (Cronbach’s *α* = 0.80–0.89). Significant differences between units with and without a psychologist further support construct validity and indicate that the PHQ is sensitive to contextual variation in how psychological expertise is valued.

Beyond its psychometric validation, the PHQ revealed a pattern of perceptions that highlights the growing recognition of psychological expertise within hospital care. Among the three domains, support and training for staff emerged as the most highly valued function across department and unit types. This finding aligns with the increasing recognition that healthcare workers’ psychological well-being is an essential determinant of quality of care (Shanafelt et al., 2019; West et al., 2016). In this respect, participants emphasized the importance of psychological support for promoting staff well-being (89.9%) and managing stress (87.0%). These perceptions are also consistent with national guidance that identifies staff support as one of the core functions of hospital psychologists (Ministry of Health, 2022a, 2022b).

Staff also attributed high importance to the psychologist’s role in supporting patients and their families, particularly in contexts involving serious conditions. The emphasis on family support—higher than in some previous studies (Palmer et al., 2025; Tomai et al., 2017)—may reflect the centrality of family caregiving in Italy, where informal care rates exceed the European average (ISTAT, 2019). These results suggest the usefulness of introducing psychoeducational interventions in hospitals to reduce the caregivers’ burden in severe illnesses (Magliano et al., 2005, 2021) together with providing psychological support to patients focusing on adjustment to illness (recommended by 80.0% of respondents). Recognition of psychologists as key figures in supporting patients and their families was higher among respondents with less work experience. This suggests that the new generation of professionals has been trained at university to consider psychological aspects as an integral part of care (Gil et al., 2023; Napolitano et al., 2024).

Consistent with the literature (Kidd & Styron, 2020; Palmer et al., 2025; Tomai et al., 2017), the role of the psychologist as an integrated team member was the least acknowledged. Specifically, only 57.3% of participants were convinced or strongly convinced that psychologists should be involved in developing care plans, and just over half recognized their role in promoting healthy lifestyles or connecting with community associations. This limited recognition could in part be explained by the prevailing staff adherence to a biomedical model of care and in part to the limited availability of psychologists in hospital settings. In Italy, psychologists account for just 1.1% of public health service healthcare staff and 75% of psychologists work in community-based facilities (Italian Ministry of Health, 2022a, 2022b). As reported by Palmer (2025), limited availability is one of the main reasons for the underutilization of this professional figure in multidisciplinary teams.

Findings revealed that the supportive role of the psychologist for patients and families was more highly valued in oncology and in medical units. This may be related both to the specific nature of the conditions treated and to more frequent exposure to psychological support in medical contexts. In oncology, the complexity of patient care and the psychological impact of the disease on patients and their families may account for the heightened recognition of psychological support needs (Alam et al., 2020). In medical units, continuity of care through outpatient follow-up may explain the greater relevance of psychological support compared with surgical and support service units. The capacity to capture differences across units suggests that the PHQ may help administrators monitor changes following new staffing models or interventions aimed at strengthening staff support or patient-centered care.

These findings can also be interpreted through implementation science frameworks. According to the Consolidated Framework for Implementation Research (CFIR; Damschroder et al., 2009), staff perceptions reflect two key domains: (a) Characteristics of Individuals, specifically their knowledge and beliefs about the “intervention,” which in this study corresponds to the psychologist’s contribution within hospital care and (b) Inner Setting, including cultural features and implementation climate. Units where psychologists were present displayed a more favorable attitude, indicating that organizational culture is a crucial driver of acceptance, while limited recognition in other units likely reflects the predominance of biomedical models or low expectations for psychosocial collaboration—consistent with previous qualitative findings in Italian hospitals (Tomai et al., 2017).

Through this lens, the PHQ emerges not only as a psychometric tool but as a potential diagnostic instrument capable of identifying organizational facilitators and barriers to the implementation of psychological services. Furthermore, although this study did not directly evaluate service changes, it yielded an unexpected practical observation: the hospital psychologist reported an increase in requests for psychological consultations after the survey was introduced. These requests concerned both clinical consultations for patients—documented in the medical record and, in some cases, reimbursed under regional tariff systems—and staff support activities, which are not reimbursed as individual services but fall within institutional functions of prevention and staff well-being. While anecdotal, this increase suggests that completing the PHQ may have raised awareness of the psychologist’s role among hospital staff.

### Strengths and Limitations

A key strength of this study is the development and psychometric validation of the Psychologist in Hospital Questionnaire (PHQ), the first tool specifically designed to assess healthcare staff’s perceptions of psychologists’ roles in Italian hospital settings. The PHQ’s brevity, ease of administration, and close alignment with national guidelines make it suitable for routine service evaluation, cross-setting comparisons, and longitudinal monitoring. The inclusion of an open-ended question to collect comments allowed the identification of specific situations not captured by the multiple-choice items. Although the main aims of this study were psychometric, the data also provide a first quantitative “snapshot” of how a broad range of hospital professionals—not only physicians and nurses but also allied health staff and healthcare assistants—conceptualize the psychologist’s contribution within hospital care.

Several limitations should be noted. Despite its strong initial psychometric performance, the PHQ is a newly developed instrument and requires further testing. The present validation relies on a single sample from one institution, and cross-validation in other samples is needed. Moreover, while the Italian version underwent psychometric evaluation, no English version of the PHQ has yet been validated, limiting its immediate international applicability. The instrument captures exclusively self-reported perceptions, which may be affected by social desirability bias and may not fully reflect actual clinical practices or patterns of interdisciplinary collaboration. The sample, although larger than those of previous studies on this topic (Tomai, 2017, p. 15 clinicians; Palmer et al., 2025, p. 49 clinicians; LaGrotte et al., 2024, p. 77 psychologists), represented 21% of the hospital workforce (AORN, 2024) and came from a single hospital in Southern Italy—a region characterized by lower healthcare resource availability compared to other parts of the country (SVIMEZ, 2024). This restricts the generalizability of the findings.

In addition, the organization and funding of psychological services in Italy vary considerably across regions and health trusts. Clinical psychological interventions documented in the medical record are often reimbursed by regional health systems, whereas activities such as staff support, training, and interdisciplinary consultation are typically financed through hospital or regional budgets and are not reimbursable as individual billable services (Ministry of Health, 2022a, 2022b; CNOP, 2017). These differences may influence the visibility of psychologists in daily practice and, consequently, staff perceptions captured by the PHQ. Future research should replicate the validation of the instrument in other hospitals and regions, test measurement invariance across settings, and examine test–retest reliability and convergent/discriminant validity. It should also examine how different organizational models of psychological service delivery shape perceptions and integration of psychologists within healthcare teams.

Overall, the PHQ demonstrates strong psychometric properties and offers a concise and reliable measure of hospital staff’s perceptions of psychologists’ roles. By capturing differences across departments and organizational contexts, it may support hospitals in monitoring the integration of psychological services and identifying areas where collaboration and support could be strengthened.

## Data Availability

The data that support the findings of this study are available from the corresponding author upon reasonable request. Requests must include a protocol and statistical analysis plan, and must not conflict with the publication plan.
